# Feeling hopeful: development and validation of the trait emotion hope scale

**DOI:** 10.3389/fpsyg.2024.1322807

**Published:** 2024-01-19

**Authors:** David B. Feldman, Hooria Jazaieri

**Affiliations:** ^1^Department of Counseling Psychology, Santa Clara University, Santa Clara, CA, United States; ^2^Leavey School of Business, Santa Clara University, Santa Clara, CA, United States

**Keywords:** hope, emotion, scale development, scale construction, affect, Hope Theory

## Abstract

While prominent measures of hope are largely cognitive in nature, many scholars and laypeople view hope primarily as an emotion. Although Snyder’s Elaborated Hope Theory attempts to theoretically balance these two perspectives, no measure yet exists of hope as a purely emotional process, only as a cognitive process. Overlooking the emotional features of hope limits our ability to more fully and precisely understand this construct. As such, across three studies (*N* = 2,900), we develop and validate the Trait Emotion Hope Scale (TEHS). In Study 1, we report on item development and piloting of the TEHS, examining internal consistency as well as convergent and discriminant validity. Study 2 includes an exploratory factor analysis (EFA) and further examines internal consistency and construct validity. Finally, in Study 3 we report a confirmatory factor analysis (CFA) to cross-validate the factor structure identified in Study 2 in a large, international sample. Importantly, we find that the TEHS accounts for significant unique variance beyond cognitive hope, indicating that the two constructs are distinct and not redundant. Taken together, these three studies demonstrate that the TEHS is psychometrically sound and provides a valid measure for those interested in examining hope as an emotion in their research.

## Introduction

During trying times across the globe, hope is of utmost relevance. The literature on hope has flourished over the past few decades (for meta-analyses see Reichard et al., 2013; Marques et al., 2017). While many scholars and laypeople alike consider hope to be an emotion, at least in part, the vast majority of the literature on hope has utilized self-report measures which operationalize hope in purely cognitive terms (Redlich-Amirav et al., 2018). As described by Keltner and Horberg (2015), cognition typically refers to mental processes that more deliberative and controlled, and are rooted in language, conceptual knowledge, and representation. Cognition leads to emotions in that emotions often occur when events, objects, and people are appraised and evaluated (i.e., “emotion-eliciting appraisals”). Emotions are considered to be brief, physiologically-based states that are positively or negatively valanced phenomenological experiences that serve to orient the person to respond. Although prominent theories on hope, most notably Snyder’s (2002) “Hope Theory,” support the notion that hope includes both a trait-like emotion set (i.e., disposition toward experiencing certain emotions regarding goal attainment and goal non-attainment; Snyder, 2002) as well as a cognitive set (i.e., cognitive processes a person uses to conceptualize and pursue goals, comprising of both agency and pathway thinking; Snyder, 2002), currently no measure of hope purely assessing this emotion set exists. We find this mismatch between the theoretical conceptualization, lay perception, and operationalization of hope to be problematic. Treating (and measuring) hope as cognitive in nature and largely overlooking the emotional feature limits our ability to more fully and precisely understand this construct.

To this end, in this article, we detail the development and validation of the Trait Emotion Hope Scale (TEHS). In particular, the TEHS was designed to assess hope as a trait-like “emotion set” as described in Snyder’s (2002) Elaborated Hope Theory. In a series of three studies, we test the internal consistency, criterion validity, and factor structure of the TEHS. First below, we briefly review the general literature on hope as both cognition and emotion, particularly highlighting Hope Theory and the role of the emotion set in the Elaborated Hope Model (Snyder, 2002). We also describe current prominent measures of hope and the development of the TEHS.

## Hope as cognition - hope theory

Over the past three decades, C. R. Snyder’s conceptualization of hope has received considerable attention (Snyder, 1994, 2002). The original version of this model, known as Hope Theory (Snyder et al., 1991), conceptualized hope as a cognitive, goal-directed construct, where goals were defined as anything an individual desires to get, do, be, experience, or accomplish. Thus, virtually all behavior can be considered as being directed toward achieving some goal (Snyder, 1994; Snyder et al., 1999).

Within this context, Snyder et al. (1991) further defined hope as “a cognitive set that is based on a reciprocally-derived sense of successful agency (goal-directed determination) and pathways (planning to meet goals)” (p. 571). Thus, hope is comprised of two additional components, pathways thinking and agency thinking. A pathway is considered to be a cognitive route to a goal (Snyder, 1994). As such, people engage in “pathways thinking” whenever they make plans regarding how to reach their goals. Agency thinking is defined as “the thoughts that people have regarding their ability to begin and continue movement on selected pathways toward those goals” (Snyder et al., 1999, p. 180). Agency thoughts provide individuals with the motivation to pursue their goals (Snyder et al., 1998).

Thus, within the original version of Hope Theory, hope was not conceptualized as an emotion. Nonetheless, the combination of these three cognitive elements (goals, pathways, and agency) was theorized to give rise to emotion, or an “emotion set.” In particular, Snyder (1994) theorized that, when people believe that their goals are blocked or not possible to achieve (i.e., low levels of pathways and agency thinking), negative emotions result. In contrast, when people believe that they are likely to achieve their goals (i.e., high levels of pathways and agency thinking), positive emotions result. Although Snyder did not originally conceptualize hope as an emotional experience, hope has been shown to have implications for affect more generally (e.g., Feldman and Snyder, 2005; Vacek et al., 2010; Ritschel and Sheppard, 2018).

## Hope as emotion

In contrast to the model described above, many consider hope to be primarily an emotion (e.g., Averill et al., 1990; Aspinwall and Leaf, 2002; Emmons, 2005; Scioli et al., 2011). In particular, some argue that while goal-related cognitions can be sources of hope, they are not necessarily hope in itself. Instead, these scholars assert that hope is best conceptualized as a positive emotion that accompanies, or results from, cognitions or thoughts (Hornsey and Fielding, 2016; Boukala and Dimitrakopoulou, 2017). Lazarus (1999), for instance, wrote that “hope is a response to goal outcomes, and, as such, it should be treated as an emotion” (p. 663).

In one study, Bruininks and Malle (2005) sought to understand how individuals distinguish hope from other affective states, including optimism, wanting, desire, wishing, and joy. In particular, they asked a sample of college students to qualitatively describe each of these states, particularly focusing on how they would use the word in everyday conversation. Similar to most of the other states, hope was primarily described as an emotion (56% of the time) and as a cognition only 40% of the time.

Scioli et al. (2011) developed a complex theoretical view of the emotion of hope. They defined hope as “a future-directed, four channel emotion network.” That is, rather than being a single experience, hope is an underlying network of needs and experiences known as “channels.” These authors originally hypothesized that hope would consist of four broad channels: mastery, attachment, survival, and spiritual. They then developed and refined a series of items designed to measure these hope channels in both trait and state forms. Although factor analyses supported the hypothesized four-factor structure for state hope, six factors were revealed for trait hope (attached mastery, personalized mastery, basic trust, attached survival, self-generated survival, and spirituality).

Perhaps partially in response to critiques of a completely cognitive view of hope, Snyder (2002) revised his original conceptualization of Hope Theory, producing “Elaborated Hope Theory.” This model retains its original three cognitive components (goals, pathways, and agency), but more prominently includes the role of emotions in the “emotion set.” It is upon the role of emotions in this elaborated model that we have developed the new measure we test in the subsequent series of studies. We now review the Elaborated Hope Model in detail.

## Elaborated hope model

The Elaborated Hope Theory Model (Snyder, 2002) is divided into two main temporal segments: the event sequence and learning history. The event sequence consists of the generation of pathways and agency thinking in the moment-to-moment goal-pursuit process – this can be considered the “state” segment of the model. In other words, a person might have a particular goal at a particular moment (e.g., to find a job), and thus generate the necessary pathways thoughts (i.e., plans for pursuing this particular goal, such as updating one’s resume) and agency thoughts (e.g., “I’ve gotten a job before, so I know I can do it again”) to go about pursuing that goal. The event sequence culminates in the eventual achievement (or not) of the goal. As mentioned previously, as the individual moves closer to achieving the goal, this generates positive emotions; when the individual moves further from achieving a goal or perceives that the goal is, or will be, blocked, this generates negative emotions.

Over time, such emotional states create and elaborate trait-like dispositions toward goal pursuits, which Snyder (2002) called the learning history segment of the model. This learning history segment is composed of both a cognitive and emotion set. The cognitive set involves enduring agency and pathways beliefs one has developed from past experiences with pursuing goals. The emotion set is similar, but involves “residue from myriad previous goal pursuits, such that the dispositionally high-hope person’s self-referential emotions reflect positive and active feelings about engaging in future goal pursuits” (Snyder, 2002, p. 253). These two trait-like “sets” then influence people’s future goal-pursuit processes (i.e., future event sequences). Thus, in the elaborated model, Snyder (2002) posited that emotions are not only state-like outcomes of goal-directed cognitive processes, but also form a trait-like hopeful emotion set, which can *influence* future goal-directed cognitive processes.

Measures have been developed to assess the cognitive elements of both segments of the elaborated model. With regard to the event sequence segment, the State Hope Scale (Snyder et al., 1996) and Goal-Specific Hope Scale (Feldman et al., 2009) assess state-like changes in agency and pathways thinking as individuals pursue their goals. With regard to the learning history segment, the original Adult Hope Scale (AHS; Snyder et al., 1991), which is sometimes referred to as the “Trait” Hope Scale, was developed to assess dispositional agency and pathways beliefs. As such, it assesses the cognitive set referred to earlier.

On the other hand, no measure has yet been developed to assess the emotion set, which is the goal in the present research. Thus, in the present research, we seek to measure the role of emotions as detailed in the elaborated model. In the most complete account of this model, Snyder (2002) theorized that emotions manifest in three connected ways. First, individuals who are dispositionally high in hope should experience general or global feeling of hopefulness. For instance, Snyder (2002) theorized that “a high-hope person should have enduring positive emotions” (p. 252) and that “high-hopers’ emotions consistently are flavored with friendliness, happiness, and confidence” (p. 253). Second, people who are dispositionally high in hope should experience feelings of hopefulness regarding their goals. Snyder (2002) writes that “the high-hope person enjoys goal pursuits and pursues them with a positive emotional set” (p. 254) and that such individuals’ emotions “reflect positive and active feelings about engaging in future goal pursuits” (p. 253). Finally, individuals who are dispositionally high in hope should experience feelings of hopefulness in the face of goal-related setbacks. Specifically, Snyder theorizes that when low-hope individuals perceive goal-related derailments, the “disruptive negative emotions cycle back to register on the person’s dispositional and situational hopeful thinking” (p. 255). In contrast, when high-hope individuals perceive such derailments, they experience “approach emotions so as to reinforce the person’s dispositional and situational hopeful thinking” (p. 255). As detailed later, we developed items reflecting each of these roles for hopeful emotion in the elaborated model.

## Measures of hope

While scales based on Snyder’s (2002) Hope Theory are the most widely used in the literature (Redlich-Amirav et al., 2018), other measures are also used such as the Herth Hope Scale (HHS; 1991) and the Herth Hope Index (HHI; Herth, 1992). Although Snyder’s measures are common in the psychological and organizational behavior literatures, the HHS and HHI are prominent in the nursing and medical literatures. Factor analyses indicated that the HHS and HHI instruments tap three (mostly cognitive) dimensions: (1) temporality and future, (2) readiness and expectancy, and (3) interconnectedness (inter- and intra-personally) (Herth, 1991, 1992). Subsequent studies, however, have yielded two factors: (1) future-focused expectancy, and (2) interconnectedness (Nayeri et al., 2020).

Another widely used measure in psychological research is the Beck Hopelessness Scale (BHS; Beck et al., 1974). While the items of the BHS are summed to reflect a total hopelessness score, an initial factor analysis identified three dimensions – future expectations, loss of motivation, and feelings about the future. Beck et al. (1974) stated that “the underlying assumption is that hopelessness can be readily objectified by defining it as a system of cognitive schemas whose common denomination is negative expectations about the future” (p. 864). Thus, the BHS also operationalizes the construct in a largely cognitive manner.

Krafft et al. (2019) developed the Perceived Hope Scale (PHS) based partially on hope/optimism items drawn from the World Health Organization Quality of Life Spirituality, Religion and Personal Beliefs Questionnaire (WHOQOL-SRPB). Its short length (6 items) renders it easy to use, which offers an advantage relative to other hope scales. Moreover, it has been shown to have strong psychometric properties, including across cultures (Krafft et al., 2021; Marujo et al., 2021). Although it was not specifically developed to assess hope exclusively as an emotion, the PHS contains items including “I feel hopeful.” It also contains items that appear to be somewhat more cognitive in nature (e.g., “My hopes are usually fulfilled”) as well as an item regarding the effects of hope (“Hope improves the quality of my life”). Nonetheless, it should be noted that factor analysis demonstrates that it assesses a unitary construct (Krafft et al., 2019).

A somewhat less-used measure was developed by Scioli et al. (2011) based on the multidimensional, four channel emotion network model of hope detailed earlier. Their scale development process produced two measures: The Comprehensive State Hope Scales (CHS-S) consists of 40 items divided into 10 subscales (and 4 larger “channels”). The Comprehensive Trait Hope Scales (CHS-T) consists of 56 items divided into 14 subscales (and 6 larger “channels”). Given that these scales measure an “emotion network” rather than a single emotional experience, however, many of their items seem more cognitive or behavioral in nature than strictly affective (e.g., “I can handle any current or future difficulties,” “I’m succeeding in ways that really matter to me,” “I’m capable of finding support from others when I need it.”)

As can be seen, prominent measures of hope, including those mentioned in this section as well as those based on Snyder’s (2002) Hope Theory, are often somewhat cognitive in nature. Thus, these measures do not fully address the many scholarly and lay interpretations of hope as an emotion. Moreover, none were designed to assess the “emotion set” component of Snyder’s (2002) Elaborated Hope Model, a prominent theory of hope within the literature, as mentioned. This further bolsters the potential usefulness of developing a measure of this emotion set.

## TEHS item development

We developed the Trait Emotion Hope Scale (TEHS) to measure hope as an emotion and, more particularly, to assess the “emotion set” outlined in Elaborated Hope Theory (Snyder, 2002). The process of constructing the initial scale items was as follows: We began by brainstorming candidate items based on a close reading of Snyder’s (2002) paper, which details the role of the emotion set in the elaborated model. As detailed previously, this emotion set appeared to manifest in three ways within this model, as a disposition for feeling: (1) the general or global feeling of hopefulness, (2) hopeful about one’s goals, and (3) hopeful in the face of goal-related setbacks. In the initial brainstorming process, we generated dozens of items to possibly tap each of these three broad themes. Next, we examined this initial pool of items and eliminated those that were redundant or unclearly worded, resulting in a set of 15 items (see Table 1), with five items assessing each of the three themes described above. Our intention in developing items related to these themes was not necessarily to construct three subscales tapping independent constructs, but rather to comprehensively assess the role of the emotion set as detailed in the elaborated hope model.

**TABLE 1:** Initial items of the Trait Emotion Hope Scale (TEHS).

Item	Theme	Item
1*	GHA	In general, I feel filled with hope.
2*	GHA	Most days, I feel full of hope.
3	GHA	On a typical day, I feel hopeful.
4R	GHA	I often feel hopeless.
5R	GHA	Most days, I don’t feel hopeful.
6*	GDHA	I feel hopeful when I think about my goals.
7	GDHA	Whenever I consider pursuing a goal, I feel full of hope.
8*	GDHA	I feel hopeful about getting the things in life that are most important to me.
9R	GDHA	When I think about my goals, I often feel hopeless.
10R	GDHA	I feel hopeless whenever I consider pursuing goals.
11*	RHA	When things don’t go my way, I still feel hopeful.
12	RHA	Even if others get discouraged, I feel hopeful.
13*	RHA	Even when I experience setbacks, I still feel hopeful.
14R	RHA	When I face setbacks, I often feel hopeless.
15R	RHA	When things don’t go my way, I often fall into hopelessness.

R, Reverse coded item; *, included in the final TEHS scale, GHA, General Hopeful Affect; GDHA, Goal-Directed Hopeful Affect; RHA, Resilient Hopeful Affect.

## Present research

Across three studies, we report on the validation of the TEHS. Study 1 reports on the item development and piloting of the TEHS, focusing on internal consistency reliability and convergent and discriminant validity of an initial 15-item version of the scale. Study 2 further tests internal consistency and criterion validity and includes an exploratory factor analysis (EFA) to reduce the number of scale items (to a 6-item version) and to identify a statistically meaningful factor structure for the scale. Finally, Study 3 uses a confirmatory factor analysis (CFA) to assess the replicability of the factor structure of the 6-item version of the scale identified in Study 2 in a large, international sample. All three studies use distinct participant samples in an effort to address the generalizability of the scale across various populations. In all three studies, participants provided informed consent (via an online form) in accordance with the recommendations of the institutional review board. See Table 2 for a summary of the three studies in this paper.

**TABLE 2:** Summary of the three studies in this paper.

Study	Aims	TEHS version	Sample	Scales
1	Examine internal consistency reliability; convergent and discriminant validity	15-item	182 U.S. undergraduate students	∙Adult Hope Scale ∙Anxiety ∙Alexithymia ∙Cognitive Reappraisal ∙Depression ∙Dispositional Positive Emotions ∙Expressive Suppression ∙General Self-Efficacy ∙Grit ∙Happiness ∙Internal Locus of Control ∙Negative Affect ∙Optimism ∙Personal Mastery ∙Positive Affect ∙Positive Emotional Intensity and Positive Emotion Duration ∙Resilience ∙Satisfaction with Life ∙Sense of Perceived Constraints ∙Stress
2	Further test internal consistency and criterion validity; conduct exploratory factor analysis (EFA) to reduce the number of scale items to identify a statistically meaningful factor structure	15-item	542 U.S. adults	∙Adult Hope Scale ∙Anxiety ∙Depression ∙Dispositional Positive Emotions ∙Hopelessness ∙Personal Mastery ∙Positive Emotional Intensity and Positive Emotion Duration ∙Self-consciousness ∙Sense of Perceived Constraints ∙Social Desirability ∙Stress
3	Conduct confirmatory factor analysis (CFA) to assess the replicability of the factor structure identified in Study 2 in an international sample	6-item	2,176 adults from 193 countries around the globe	∙Happiness ∙Interpersonal Generosity ∙Loneliness ∙Personality ∙Flourishing ∙Resilience ∙Sense of Power ∙Stress ∙Values

## Study 1

The purpose of this study was to pilot test the TEHS to examine scale reliability as well as convergent and discriminant validity via correlations with established scales. In terms of convergent validity, we expected that the TEHS would be significantly positively related to a variety of measures. First, we expected the TEHS to be related to the Adult Hope Scale (Snyder et al., 1991), which assesses the cognitive set within Snyder’s (2002) Hope Theory. Second, we selected convergent measures assessing constructs theoretically related to hope, including optimism (Scheier et al., 1994), general self-efficacy (Schwarzer and Jerusalem, 1995), internal locus of control (Rotter, 1966), personal mastery (Lachman and Weaver, 1998), and grit (Duckworth and Quinn, 2009). Third, we selected convergent measures assessing other positive emotions, including joy, contentment, pride, love, compassion, amusement, and awe (Shiota et al., 2006), positive and negative affectivity (Watson et al., 1988), and positive emotional intensity and positive emotion duration (Becerra et al., 2019). Finally, we selected convergent measures that have shown strong relationships with measures of cognitive hope in past research (e.g., Bailey and Snyder, 2007; Halperin and Gross, 2011; Morote et al., 2017; Peh et al., 2017; Witvliet et al., 2019), including: resilience (Smith et al., 2008), cognitive reappraisal (Gross and John, 2003), satisfaction with life (Diener et al., 1985), and happiness (Lyubomirsky and Lepper, 1999).

In terms of discriminant validity, we similarly selected measures that have been shown to have inverse relationships with measures of cognitive hope in past research (e.g., Sears and Kraus, 2009; Peh et al., 2017; Feldman, 2021; Wang et al., 2022). Thus, we expected that the TEHS would be significantly inversely related to measures of other negative emotions (Watson et al., 1988; Lovibond and Lovibond, 1995), expressive suppression (Gross and John, 2003), sense of perceived constraints (Lachman and Weaver, 1998), and identifying and describing feelings (Bagby et al., 1994).

### Participants and procedures

Participants were undergraduate students (*N* = 200) from a private university in the United States who self-enrolled into the study for partial course credit. Eighteen participants were removed from the study for missing two or more (out of four) attention checks, leaving a final sample of 182 participants. Participants were primarily women (65.4%), White (55%), and M_*age*_ 19.01(*SD* = 1.42). This sample size exceeds the recommended minimum (*N* = 30) for scale piloting (Johanson and Brooks, 2009).

Following informed consent, participants completed, in randomized order, all of the measures described in the subsequent section. At the end, participants completed a demographics questionnaire.

### Measures

#### Trait emotion hope scale (TEHS)

As described previously, the TEHS was developed based on the prior theoretical literature on hope which informed an initial set of 15 items. The 15 items (presented in random order) were rated on an 8-point Likert scale (1 = *Definitely False*, 8 = *Definitely True*). The total score is derived by summing all items, with higher scores indicating greater emotional hope (See Table 1 for the full original scale). Internal consistency is reported in the Results section.

#### Adult hope scale (AHS)

The AHS (Snyder et al., 1991) measures Snyder’s cognitive model of hope (Snyder, 1994). The scale contains 12 items (four measuring pathways thinking, four measuring agency thinking, and four serving as fillers) rated on an 8-point Likert scale (1 = *Definitely False*, 8 = *Definitely True*). The total AHS score is derived by summing the four agency and the four pathway items, with higher total scores indicating greater hope. Internal consistency was α = 0.87.

#### Brief resilience scale (BRS)

The BRS (Smith et al., 2008) is a 6-item measure of resilience. Each item is rated on a 5-point Likert scale (1 = *strongly disagree*, 5 = *strongly agree).* All items are summed and divided by six for an average resilience score, with higher scores indicating greater resilience. Internal consistency was α = 0.86.

#### Depression, anxiety, stress scale (DASS-21)

The DASS-21 (Lovibond and Lovibond, 1995) consists of three subscales measuring depression, anxiety, and stress. The 21 items (seven for each subscale) are rated on a 4-point Likert scale (0 = *does not apply to me at all*, 3 = *applied to me very much or most of the time*). Scores for each subscale are calculated by summing the scores of the relevant items, with higher scores indicating greater symptoms. Internal consistency was as follows – depression: α = 0.89; anxiety: α = 0.83; and stress: α = 0.83.

#### Dispositional positive emotion scale (DPES)

The DPES (Shiota et al., 2006) is a 38-item instrument with seven subscales rated on a 7-point Likert scale (1 = *strongly disagree*, 7 = *strongly agree*). The seven emotions measured are: joy, contentment, pride, love, compassion, amusement, and awe. Each subscale is separately summed, with higher scores reflecting greater positive emotion. Internal consistency was as follows – joy: α = 0.84; contentment: α = 0.89; pride: α = 0.74; love: α = 0.79; compassion: α = 0.82; amusement: α = 0.79; and awe: α = 0.78.

#### Emotion regulation questionnaire (ERQ)

The 10-item ERQ (Gross and John, 2003) measures two emotion regulation strategies of expressive suppression (4 items) and cognitive reappraisal (6 items). Items are rated on a 7-point Likert-type scale (1 = *strongly disagree*, 7 = *strongly agree*). Ratings are averaged for each subscale, with higher scores reflecting greater endorsement of the strategy. Internal consistency was as follows – expressive suppression: α = 0.75; cognitive reappraisal: α = 0.78.

#### General self-efficacy scale (GSE)

The GSE (Schwarzer and Jerusalem, 1995) is a 10-item scale of self-efficacy rated on a 4-point Likert scale (1 = *Not at all true*, 4 = *Exactly true*). The scale is summed, with higher scores indicating greater self-efficacy. Internal consistency was α = 0.87.

#### GRIT-short (GRIT-S)

The 8-item GRIT-S (Duckworth and Quinn, 2009) is designed to measure trait-level perseverance and passion for long-term goals. Items are rated on a 5-point Likert scale (1 = *not like me at all*, 5 = *very much like me*) and are summed and divided by 8 to yield an average score, where higher scores indicating greater grit. Internal consistency was α = 0.83.

#### Life orientation test revised (LOT-R)

The LOT-R (Scheier et al., 1994) is a 10-item measure (with four filler items) of dispositional optimism. All items are rated on a 4-point Likert scale (0 = *strongly disagree*, 4 = *strongly agree*). After reversing the appropriate items, ratings are summed to yield a total score, with higher scores indicating greater optimism. Internal consistency was α = 0.82.

#### Perth emotional reactivity scale: positive intensity and duration (PERS)

The PERS (Becerra et al., 2019) is a self-report measure consisting of six 5-item scales of emotional reactivity. The PERS measures activation, intensity, and duration of both negative and positive emotions. In the present research we were interested in measuring only positive intensity (5 items) and positive duration (5 items). Items are rated on a 5-point Likert scale (1 = *very unlike me*, 5 = *very like me*). Each subscale is summed, with higher scores representing higher levels of reactivity. Internal consistency was as follows – positive intensity: α = 0.88; positive duration: α = 0.88.

#### Positive and negative affect schedule (PANAS)

The 20-item PANAS (Watson et al., 1988) measures 10 positive affective states and 10 negative affective states using a 5-point Likert scale (1 = *very slightly/not at all*, 5 = *extremely*). Total scores for positive and negative affect are determined by summing the ten appropriate items, with higher total scores indicating greater affect. Internal consistency was as follows – PANAS positive: α = 0.87; PANAS negative: α = 0.88.

#### Locus of control scale (LOCS)

The LOCS (Rotter, 1966) is a 29-item measure (with six filler items) of locus of control beliefs. For each item, respondents are given two alternative statement and asked to choose the one that more closely reflects their own beliefs. One point is given for each response that reflects an external locus of control with higher scores indicating an external locus of control and lower scores indicating an internal locus of control. Internal consistency was KR-20 = 0.70.

#### Satisfaction with life scale (SWLS)

The SWLS (Diener et al., 1985) is a 5-item measure of life satisfaction. Items are rated on a 7-point Likert scale (1 = *Strongly Disagree*, 7 = *Strongly Agree*) and are summed, with higher scores indicating greater life satisfaction; α = 0.85.

#### Sense of control scale (SCS)

The 12-item SCS (Lachman and Weaver, 1998) includes 4 items assessing personal mastery and 8 items assessing perceived constraints. Items are rated on a 7-point Likert scale (1 = *Strongly Disagree*, 7 = *Strongly Agree*). Internal consistency was as follows – Personal mastery: α = 0.81; Perceived constraints: α = 0.84.

#### Subjective happiness scale (SHS)

The SHS (Lyubomirsky and Lepper, 1999) is a 4-item measure of happiness. All items are rated on a 6-point Likert scale with anchors differing from item to item. After reverse scoring one item, all items are summed, with higher scores indicating greater happiness. Internal consistency was α = 0.87.

#### Toronto alexithymia scale (TAS-20)

The TAS-20 (Bagby et al., 1994) is a 20-item measure of alexithymia (difficulties identifying and describing one’s feelings). Each item is rated on a 5-point Likert scale (1 = *strongly disagree*, 5 = *strongly agree*). The TAS-20 consists of three subscales: difficulty describing feelings (5 items), difficulty identifying feelings (7 items), and externally-oriented thinking (8 items) and can be reported as a total score of all 20-items or separate scores for each subscale. Internal consistency was as follows – TAS-20 total: α = 0.85; difficulty describing feelings: α = 0.84; difficulty identifying feelings: α = 0.86; and externally-oriented thinking: α = 0.76.

#### Demographics

Participants were asked to provide basic demographic information, including age, gender, and ethnicity.

### Statistical analysis

All analyses were conducted in SPSS version 28 (IBM, 2021). Cronbach’s alpha (α) and McDonald’s omega (ω) were used to assess internal consistency reliability of the scale. Pearson’s correlations assessed convergent and discriminant validity. Basic descriptive statistics were characterized by means and standard deviations.

### Results

The 15-item TEHS had excellent internal consistency reliability, α = 0.95 (ω = 0.95). The range of corrected item-total correlations were from 0.63 to 0.84. When examining the three potential subscales, they too had very strong internal consistency, general hopeful affect: α = 0.93, goal directed hopeful affect: α = 0.88, and resilient hopeful affect: α = 0.88. The Cronbach’s Alpha if item was deleted statistic suggested that none of these subscales (or the total scale) could be further improved. Thus, all 15-items were retained for Study 2, where we attempt to further reduce the total number of items.

Means, standard deviations, and bivariate correlations are reported in Table 3. Correlational analyses revealed that the TEHS was related to constructs that we would expect, including happiness (*r* = 0.74), cognitive hope (*r* = 0.72), optimism (*r* = 0.71), life-satisfaction (*r* = 0.64), self-efficacy (*r* = 0.63), resilience (*r* = 0.61), and grit (*r* = 0.55). The TEHS was also positively related to positive emotional intensity and duration (via the PERS) and positive affect more generally (via the DPES and PANAS positive affect subscale), with the exception of the amusement subscale of the DPES which was unrelated to the TEHS (*r* = 0.09, *p* = 0.235). Those who endorsed greater TEHS also reported greater endorsement of cognitive reappraisal. Additionally, those who endorsed greater TEHS reported greater internal (as opposed to external) locus of control and greater personal mastery.

**TABLE 3:** Study 1 means, standard deviations, and bivariate correlations.

Variables	TEHS –15-item total	TEHS –GHA	TEHS –GDHA	TEHS –RHA	M (SD)
TEHS – 15-item Total	− −	0.93**	0.90**	0.90**	79.25 (18.80)
TEHS – GHA	0.93**	− −	0.76**	0.77**	26.52 (7.47)
TEHS – GDHA	0.90**	0.76**	− −	0.69**	28.49 (6.72)
TEHS – RHA	0.90**	0.77**	0.69**	− −	24.24 (6.48)
AHS – Total	0.72**	0.67**	0.71**	0.59**	46.81 (7.85)
AHS – *Agency*	0.71**	0.67**	0.70**	0.56**	23.76 (4.54)
AHS – *Pathways*	0.59**	0.53**	0.57**	0.51**	23.05 (4.20)
BRS	0.61**	0.61**	0.45**	0.60**	3.04 (0.77)
DASS-21 – *Depression*	-0.71**	-0.72**	-0.60**	-0.62**	6.30 (5.09)
DASS-21 – *Anxiety*	-0.55**	-0.54**	-0.50**	-0.46**	6.69 (4.84)
DASS-21 – *Stress*	-0.54**	-0.51**	-0.48**	-0.48**	8.11 (4.61)
DPES – Total	0.62**	0.62**	0.48**	0.57**	186.69 (27.28)
DPES – *Joy*	0.60**	0.64**	0.44**	0.54**	27.73 (6.20)
DPES – *Contentment*	0.70**	0.74**	0.54**	0.63**	22.87 (6.04)
DPES – *Pride*	0.64**	0.62**	0.57**	0.55**	24.31 (4.80)
DPES – *Love*	0.36**	0.35**	0.29**	0.35**	28.54 (6.02)
DPES – *Compassion*	0.24**	0.17**	0.25**	0.24**	28.89 (4.07)
DPES – *Amusement*	0.09	0.12	0.02	0.09	25.07 (5.22)
DPES – *Awe*	0.38**	0.38**	0.28**	0.39**	29.38 (5.69)
ERQ – *Cognitive Reappraisal*	0.43**	0.40**	0.36**	0.40**	28.24 (6.00)
ERQ – *Expressive Suppression*	-0.14	-0.14	-0.15	-0.10	14.78 (5.05)
GSE	0.63**	0.55**	0.57**	0.61**	29.43 (4.47)
GRIT-S	0.55**	0.48**	0.55**	0.47**	3.00 (0.69)
LOT-R	0.71**	0.70**	0.59**	0.64**	13.03 (4.55)
PERS – *Positive Intensity*	0.36**	0.36**	0.26**	0.36**	18.06 (4.15)
PERS – *Positive Duration*	0.50**	0.51**	0.41**	0.43**	17.96 (4.30)
PANAS – *Positive Affect*	0.67**	0.64**	0.58**	0.59**	32.11 (7.30)
PANAS – *Negative Affect*	-0.58**	-0.55**	-0.54**	-0.47**	24.54 (7.86)
LOCS	-0.40**	-0.36**	-0.40**	-0.34**	13.43 (3.70)
SWLS	0.64**	0.66**	0.50**	0.59**	22.55 (6.24)
SCS – *Personal Mastery*	0.47**	0.42**	0.48**	0.39**	22.00 (4.07)
SCS – *Perceived Constraints*	-0.67**	-0.57**	-0.61**	-0.64**	27.14 (8.75)
SHS	0.74**	0.78**	0.54**	0.68**	4.48 (1.29)
TAS-20 – Total	-0.40**	-0.35**	-0.35**	-0.38**	51.48 (11.33)
TAS-20 – *Describe*	-0.38**	-0.33**	-0.33**	-0.37**	14.72 (4.77)
TAS-20 – *Identify*	-0.25**	-0.21**	-0.22**	-0.25**	18.40 (5.94)
TAS-20 – *Externally oriented thinking*	-0.03	-0.00	-0.01	-0.09	18.37 (4.32)

** = *p* < 0.01. M, Mean; SD, Standard Deviation; GHA, General Hopeful Affect; GDHA, Goal-Directed Hopeful Affect; RHA, Resilient Hopeful Affect.

The TEHS was inversely related to negative affect via the DASS-21 and PANAS negative as well as perceived constraints sense of control and difficulties describing and identifying feelings. However, there was no relationship between TEHS and externally-oriented thinking (*r* = −0.03, *p* = 0.70). The TEHS was marginally inversely related to expressive suppression (*r* = −0.14, *p* = 0.06).

### Brief discussion

Study 1 set out to pilot test the reliability and validity of the initial 15 items of the TEHS. The TEHS had excellent reliability. When examining convergent validity, the TEHS correlated significantly and positively with similar constructs, including cognitive hope and positive affect. There was a range in the degree to which the TEHS related to specific positive emotions such as contentment (*r* = 0.70) versus compassion (*r* = 0.24). When examining discriminant validity, the TEHS diverged from the expected constructs such as negative affect and difficulties identifying and describing emotions. There was also a range (albeit smaller) in the inverse relationships between the TEHS and negative affective states such as depression (*r* = −0.71) and stress (*r* = −0.54). There was also a marginal inverse relationship between the TEHS and expressive suppression.

Currently, the thematic groupings (i.e., general hopeful affect, goal directed hopeful affect, and resilient hopeful affect) of the emotional experience of hope used to generate the initial pool of TEHS are still based on theory and face validity rather than psychometric methods. Thus, there is a need to psychometrically examine them. Additionally, given that single-item measures (e.g., Watson et al., 1988) and relatively short measurement tools (e.g., Shiota et al., 2006) of affect are common in the literature, some may consider an emotional hope scale with 15 items too long for practical use in psychological research and in organizational settings. Therefore, there is a need to reduce the number of items in the TEHS to arrive at a more parsimonious scale. This need motivated Study 2 and the subsequent Exploratory Factor Analysis (EFA).

## Study 2

Given that we brainstormed items for the TEHS based on three broad thematic groupings derived from Snyder’s (2002) elaborated hope theory, the primary goal of Study 2 was to psychometrically examine, refine, and reduce the items of the TEHS via Exploratory Factor Analysis (EFA) in order to arrive at the most statistically parsimonious scale. The EFA will allow us to identify any possible redundancy in the items and rule out alternative groupings of the items. As mentioned, based on the hope literature, we conceptualized that there are three types of hopeful affect (general, goal-directed, and resilient); however, it is possible that some of these items overlap such that a single grouping of emotional hope is better served rather than three separate subscales. In Study 2, we also aim to the replicate internal consistency reliability from Study 1 in a larger sample and to further examine the construct and the unique variance accounted for by the TEHS. Additionally, given the relatively high correlation between TEHS and AHS (cognitive hope) of 0.72 in Study 1, in this study, we wished to conduct multiple regression analyses to determine the degree to which each of these measures accounts for unique variance in our outcomes of interest.

For convergent validity, given the findings of Study 1, we expected the TEHS would continue to be positively related to cognitive hope (Snyder et al., 1991), other discrete positive emotions (Shiota et al., 2006), positive emotional intensity and duration (Becerra et al., 2019), and personal mastery (Lachman and Weaver, 1998). For discriminant validity, we expected the TEHS to be inversely related to negative affective states (Lovibond and Lovibond, 1995) and sense of control or perceived constraints (Lachman and Weaver, 1998). We also administered the Beck Hopelessness Scale (Beck et al., 1974), which we expected to be inversely related with the TEHS. We also wanted to examine the relationship between TEHS and self-consciousness (private, public, and social anxiety related), but we did not make any *a priori* predictions about the relationship. To examine whether respondents were engaging in impression management, we administered a widely used impression management scale (Crowne and Marlowe, 1960), which we expected to be unrelated to the TEHS. We again sought to examine whether the TEHS was accounting for significant unique variance above and beyond cognitive hope in the aforementioned measures (Snyder et al., 1991) and was therefore not redundant.

### Participants and procedures

Participants (*N* = 548) were adults located in the United States and recruited via Amazon Mechanical Turk. Six participants were removed from the study for missing two or more attention checks, leaving a final sample of 542 participants, which exceeds the recommended sample size for EFA (Beavers et al., 2013). Participants were primarily women (56.3%), White (74.4%), and M_*age*_ 40.60 (*SD* = 13.17).

Following informed consent, participants then completed all of the measures described below in random order. At the end of the survey participants completed a demographics questionnaire.

### Measures

#### Trait emotion hope scale (TEHS)

We administered the same 15-item TEHS as Study 1. Internal consistency will be reported in the Results section.

#### Adult hope scale (AHS)

We administered the same AHS (Snyder et al., 1991) as Study 1. Internal consistency was α = 0.93.

#### Beck hopelessness scale (BHS)

We administered the BHS (Beck et al., 1974), which contains 20 True/False statements regarding one’s experience during the past week. Responses are summed, with higher scores indicating higher levels of hopelessness. Internal consistency was KR-20 = 0.74.

#### Depression, anxiety, stress scale (DASS-21)

We administered the same DASS-21 (Lovibond and Lovibond, 1995) as Study 1. Internal consistency was as follows – Depression: α = 0.94; Anxiety: α = 0.87; and Stress: α = 0.90.

#### Dispositional positive emotion scale (DPES)

We administered the same DPES (Shiota et al., 2006) scale as Study 1. Internal consistency was as follows – joy: α = 0.90; contentment: α = 0.94; pride: α = 0.83; love: α = 0.86; compassion: α = 0.88; amusement: α = 0.78; and awe: α = 0.84.

#### Marlowe-crowne social desirability scale (MC-SDS)

We administered the MC-SDS (Crowne and Marlowe, 1960), which is a 33-item True/False questionnaire that assesses the degree to which respondents are responding in a socially desirable manner. Each “True” response is given one point and each “False” response is given zero points. The total score is the sum of all true statements, with higher scores indicating that participants’ answers may be influenced by the desire to “look good.” Internal consistency was KR-20 = 0.72.

#### Perth emotional reactivity scale – Positive intensity and duration (PERS)

We administered the same PERS (Becerra et al., 2019) scale as Study 1. Internal consistency was as follows – positive intensity: α = 0.92; positive duration: α = 0.92.

#### Self-consciousness scale – Revised (SCSR)

We administered the 22-item SCSR (Scheier and Carver, 1985), which measures three components of self-consciousness: private (9-items), public (7-items), and social anxiety (6-items). Items are rated on a 4-point Likert scale (3 = *a lot like me*, 0 = *not at all like me*). Higher scores indicate greater endorsement for that subscale of self-consciousness. Internal consistency was as follows – Private: α = 0.79; Public: α = 0.85; Social Anxiety: α = 0.86.

#### Sense of control scale (SCS)

We administered the same SCS (Lachman and Weaver, 1998) as Study 1. Internal consistency was as follows – Personal mastery: α = 0.91; Perceived constraints: α = 0.92.

#### Demographics

We collected demographic information including age, gender, and ethnicity.

### Statistical analysis

All analyses were conducted in SPSS version 28 (IBM, 2021). Cronbach’s alpha and McDonald’s omega (ω) were used to assess internal consistency reliability of the TEHS. Pearson’s correlations assessed convergent and discriminant validity. Basic descriptive statistics were characterized by means and standard deviations. We conducted EFA using a principal axis factoring extraction method. Negatively worded items were reverse scored before being entered into the EFA. We also conducted separate regression analyses to assess the unique variance accounted for by the TEHS beyond the existing cognitive hope measure (i.e., the AHS).

For the EFA, we took an iterative approach to item reduction. First, based on our theoretical conceptualization of the three different types of emotional hope (general, goal-directed, and resilient), we initially tried to force a three-factor solution (principle axis extraction with promax rotation). We found that three factors had significant cross loadings and therefore factors 2 and factors 3 were uninterpretable. Given this, based on the Kaiser criterion (number of extracted factors with eigenvalues above 1; Costello and Osborne, 2005), scree plot (which indicated that the number of factors was indeed a single factor occurring before the “bend in the elbow”; Fabrigar et al., 1999), and variance extraction method (number of extracted factors that explain at least 75% of the variation in the item responses; Beavers et al., 2013), we arrived at a single-factor solution. Following others (e.g., Kilby et al., 2022), given the limitations of each method available to determine the number of factors to extract, we used this combination of methods (Kaiser criterion, scree plot, and variance extraction) to triangulate the number of factors.^1^

### Results

#### Exploratory factor analysis

**FIGURE 1:**
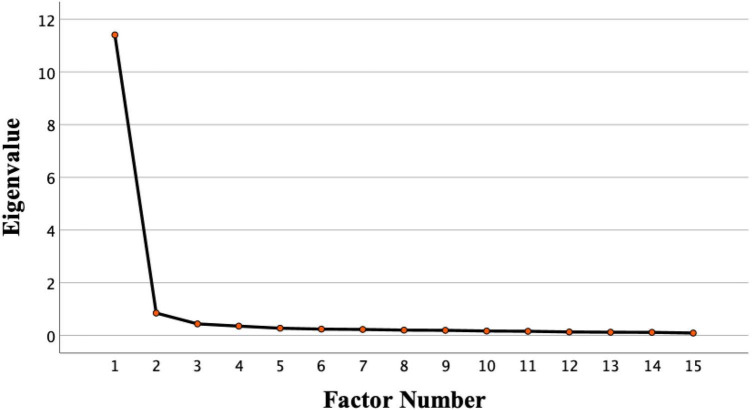
Study 2 scree plot indicating a single factor with an eigenvalue above 1.

As mentioned, in applying the EFA, all criteria for establishing the relevant number of factors converge to a clear one-factor solution. When forcing a three-factor solution, the first factor explained 76.05% of the variance (eigenvalue 11.41), the second one 5.68% (eigenvalue 0.85), and the third one 2.93% (eigenvalue 0.44). Thus, only one factor had an eigenvalue >1 (Kaiser criterion) and accounted for more than 75% of the variation in the item responses. The scree plot, which also indicated a single factor, is provided in (Figure 1). All item loadings are reported in Table 4. The 15-items had an excellent internal consistency reliability of α = 0.98^2^ (ω = 0.98), suggesting high content overlap between the 15 items. Thus, given the very high reliability of the 15-items, in order to construct the most parsimonious scale, we took the top two loading items from each of the three areas of general hopeful affect, goal-directed hopeful affect, and resilient hopeful affect (factor loadings ranging from 0.909 to 0.857) to arrive at a single-factor, 6-item scale.

**TABLE 4:** Study 2 factor loadings of TEHS items on a single-factor solution.

Item	Loading
Item 1*	0.909
Item 2*	0.900
Item 3	0.897
Item 4R	0.897
Item 5R	0.891
Item 6*	0.859
Item 7	0.856
Item 8*	0.857
Item 9R	0.848
Item 10R	0.769
Item 11*	0.863
Item 12	0.821
Item 13*	0.893
Item 14R	0.838
Item 15R	0.827

R, Reverse coded item. *= included in the final TEHS scale.

It should be noted that the top two loadings from each of these three content areas were not identical to the top six loadings from the 15-items overall (as indicated in Table 4, those items factor loadings ranged from 0.909 to 0.891). Even though we were no longer thinking of the scale as containing three separate factors, based on theorizing by Snyder (2002) regarding the “emotion set” in his elaborated hope model, we nonetheless wished to retain items across all three general areas. The intention was to maintain as much of the original theorizing as possible in the content of the items, given that the objective was to develop an assessment of this portion of Snyder’s theory. Of note, Snyder (2002) did not assert that these would necessarily be three separate factors, but was theorizing regarding how the “emotion set” works in a more general sense. So, it is not surprising that we did not find three separate factors here.

#### Internal reliability and construct validity

Table 5 contains means, standard deviations, and bivariate correlations. The 6-item TEHS had excellent internal consistency reliability, α = 0.96. The Cronbach’s Alpha if item was deleted statistic suggested that the scale could not be further improved.

**TABLE 5:** Study 2 means, standard deviations, and bivariate correlations.

Variables	TEHS 6-item r, p	M (SD)
TEHS – 6-item	− −	33.45 (9.92)
AHS – Total	0.82**	46.66 (10.22)
AHS – *Agency*	0.82**	22.89 (5.97)
AHS – *Pathways*	0.72**	23.76 (4.87)
BHS	-0.21**	9.44 (1.91)
DASS-21 – *Depression*	-0.69**	4.62 (5.56)
DASS-21 – *Anxiety*	-0.37**	2.86 (3.92)
DASS-21 – *Stress*	-0.47**	5.26 (4.81)
DPES – Total	0.78**	158.4 (33.52)
DPES – *Joy*	0.76**	26.96 (7.66)
DPES – *Contentment*	0.81**	23.59 (7.20)
DPES – *Pride*	0.76**	24.11 (5.78)
DPES – *Love*	0.57**	27.39 (7.45)
DPES – *Compassion*	0.31**	27.49 (5.25)
DPES – *Amusement*	0.24**	22.90 (5.83)
DPES – *Awe*	0.62**	28.87 (6.82)
MC-SDS	-0.04	18.60 (3.27)
PERS – *Positive Intensity*	0.46**	16.66 (4.98)
PERS – *Positive Duration*	0.68**	18.40 (4.69)
SCSR – *Private*	-0.03	15.82 (5.12)
SCSR – *Public*	-0.18**	12.00 (4.85)
SCSR – *Social Anxiety*	-0.42**	10.36 (4.86)
SCS – *Personal Mastery*	0.60**	21.03 (5.39)
SCS – *Perceive Constraints*	-0.65**	25.46 (10.68)

** = *p* < 0.01, M, Mean; SD, Standard Deviation.

Correlational analyses revealed that the 6-item TEHS was significantly positively related to the expected constructs including: cognitive hope (*r* = 0.82), positive emotion (*r* = 0.78 [DPES total]; discrete emotion ranges: *r* = 0.24 [amusement] to *r* = 0.81 [contentment]), positive emotional intensity (*r* = 0.46), positive emotion duration (*r* = 0.68), and personal mastery (*r* = 0.60).

The 6-item TEHS was also significantly inversely related to the expected constructs including: hopelessness (*r* = 0.21), depression (*r* = −0.69), anxiety (*r* = −0.37), stress (*r* = −0.47), and sense of perceived constraints (*r* = −0.65). While we did not make any *a priori* predictions, the TEHS was inversely related to public self-consciousness (*r* = −0.18) and social anxiety (*r* = −0.42), but unrelated to private self-consciousness (*r* = −0.03). As expected, there was no relationship between the TEHS and impression management (*r* = −0.04).

#### Unique variance

Given the correlation between the TEHS and AHS, we wanted to examine the degree to which each of these measures accounted for unique variance in our outcomes of interest. Linear regression analyses (Table 6) showed that the TEHS did account for unique variance over and above cognitive hope (AHS) on most of our dependent variables, with the exception of amusement. For some outcomes of interest, only the TEHS uniquely accounted for significant variance (i.e., anxiety, stress, and public self-consciousness). Interestingly, for private self-consciousness, while both cognitive hope and emotional hope accounted for significant unique variance, the relationships were in opposite directions, further indicating specificity between these two constructs. When examining hopelessness, both cognitive hope and emotional hope were marginally related (*p*’s ≤ 0.15).

**TABLE 6:** Results of regression analyses to determine unique variance accounted for by TEHS compared to cognitive hope (AHS).

					95% *CI*	
	*B*	*SE*	*t*	*p*	Lower	Upper
**BHS: *F*(2,538) = 14.90, *p* < 0.001, *R*^2^ = 0.05**						
AHS	−0.03	0.01	−0.13	0.07	−0.05	0.01
TEHS	−0.02	0.01	−1.45	0.15	−0.05	0.01
**DASS-21** – **Depression: *F*(2,538) = 256.81, *p* < 0.001, *R*^2^ = 0.49**						
AHS	−0.11	0.03	−3.65	0.001	−0.17	−0.05
TEHS	−0.30	0.03	−9.75	0.001	−0.36	−0.24
**DASS-21** – **Anxiety: *F*(2,538) = 45.76, *p* < 0.001, *R*^2^ = 0.15**						
AHS	0.01	0.03	0.22	0.82	−0.05	0.06
TEHS	−0.16	0.03	−5.64	0.001	−0.21	−0.10
**DASS-21** – **Stress: *F*(2,538) = 82.41, *p* < 0.001, *R*^2^ = 0.24**						
AHS	−0.01	0.03	−0.27	0.79	−0.07	0.05
TEHS	−0.23	0.03	−7.10	0.001	−0.29	−0.17
**DPES** – **Total: *F*(2,538) = 515.80, *p* < 0.001, *R*^2^ = 0.66**						
AHS	1.30	0.15	8.99	0.001	1.02	1.59
TEHS	1.52	0.15	10.20	0.001	1.23	1.81
**DPES** – **Joy: *F*(2,538) = 404.19, *p* < 0.001, *R*^2^ = 0.60**						
AHS	0.23	0.04	6.51	0.001	0.16	0.30
TEHS	0.39	0.04	10.43	0.001	0.31	0.46
**DPES** – **Contentment: *F*(2,538) = 618.41, *p* < 0.001, *R*^2^ = 0.70**						
AHS	0.24	0.03	8.18	0.001	0.18	0.30
TEHS	0.39	0.03	12.78	0.001	0.33	0.45
**DPES** – **Pride: *F*(2,538) = 542.27, *p* < 0.001, *R*^2^ = 0.67**						
AHS	0.31	0.03	12.64	0.001	0.26	0.36
TEHS	0.17	0.03	6.95	0.001	0.13	0.23
**DPES** – **Love: *F*(2,538) = 141.75, *p* < 0.001, *R*^2^ = 0.35**						
AHS	0.18	0.04	4.02	0.001	0.09	0.27
TEHS	0.28	0.05	6.03	0.001	0.19	0.37
**DPES** – **Compassion: *F*(2,538) = 27.67, *p* < 0.001, *R*^2^ = 0.09**						
AHS	0.06	0.04	1.70	0.09	−0.01	0.13
TEHS	0.10	0.04	2.74	0.006	0.03	0.18
**DPES** – **Amusement: *F*(2,538) = 26.12, *p* < 0.001, *R*^2^ = 0.09**						
AHS	0.18	0.04	4.38	0.001	0.10	0.26
TEHS	−0.01	0.04	−0.32	0.75	−0.10	0.07
**DPES** – **Awe: *F*(2,538) = 213.19, *p* < 0.001, *R*^2^ = 0.44**						
AHS	0.28	0.04	7.31	0.001	0.20	0.35
TEHS	0.19	0.04	5.01	0.001	0.12	0.27
**MC-SDS: *F*(2,537) = 0.84, *p* = 0.43, *R*^2^ = 0.003**						
AHS	0.02	0.02	0.94	0.35	−0.03	0.07
TEHS	−0.03	0.03	−1.28	0.20	−0.08	0.02
**PERS** – **Positive Intensity: *F*(2,539) = 83.10, *p* < 0.001, *R*^2^ = 0.24**						
AHS	0.14	0.03	4.38	0.001	0.08	0.20
TEHS	0.11	0.03	3.31	0.001	0.05	0.18
**PERS** – **Positive Duration: F(2,539) = 236.54, *p* < 0.001, *R*^2^ = 0.47**						
AHS	0.06	0.03	2.54	0.01	0.02	0.11
TEHS	0.27	0.03	10.22	0.001	0.22	0.32
**SCSR** – **Private: *F*(2,538) = 6.84, *p* < 0.001, *R*^2^ = 0.03**						
AHS	0.13	0.04	3.56	0.001	0.06	0.21
TEHS	−0.13	0.04	−3.50	0.001	−0.21	−0.06
**SCSR** – **Public: *F*(2,538) = 9.94, *p* < 0.001, *R*^2^ = 0.04**						
AHS	0.02	0.04	0.55	0.58	−0.05	0.09
TEHS	−0.11	0.04	−2.97	0.003	−0.18	−0.04
**SCSR** – **Social Anxiety: *F*(2,538) = 68.58, *p* < 0.001, *R*^2^ = 0.20**						
AHS	−0.13	0.03	−4.09	0.001	−0.20	−0.07
TEHS	−0.10	0.03	−2.88	0.004	−0.16	−0.03
**SCS** – **Personal Mastery: *F*(2,536) = 197.33, *p* < 0.001, *R*^2^ = 0.42**						
AHS	0.25	0.03	8.23	0.001	0.19	0.31
TEHS	0.11	0.03	3.51	0.001	0.05	0.17
**SCS** – **Perceived Constraints: *F*(2,536) = 249.30, *p* < 0.001, *R*^2^ = 0.48**						
AHS	−0.45	0.06	−7.87	0.001	−0.56	−0.34
TEHS	−0.32	0.06	−5.41	0.001	−0.44	−0.20

*B*, Unstandardized Beta Coefficient; *SE*, standard error; *CI*, confidence interval.

### Brief discussion

Study 2 set out to examine an EFA of the TEHS. Using this statistical approach, we were able to identify a parsimonious, 6-item form of the TEHS, which had excellent internal reliability. Similar to Study 1, the TEHS was significantly positively correlated with expected constructs such as cognitive hope and positive emotions, and was significantly negatively correlated with expected constructs such as hopelessness and negative affective states. Regression analyses suggests that while the TEHS and cognitive hope (AHS) are highly correlated, the TEHS is indeed accounting for significant unique variance beyond cognitive hope, indicating that the two constructs are distinct and not redundant. Given the reliance on statistical inference to arrive at this structure, a CFA is needed to confirm this factor structure in a separate sample. Furthermore, an assessment of convergent and discriminant validity is needed in a large, more diverse sample with a broader range of variables. Thus, we conducted Study 3.

## Study 3

Following the identification of the final six items of the TEHS via statistical inference in Study 2, we sought to validate the single-factor structure from the previous study in a new sample using Confirmatory Factor Analysis (CFA). We used a multinational sample of adults around the globe in order to examine generalizability. This helps ensure that the factor structure arising from the EFA in Study 2 is generalizable to adults around the world.

Continued evaluation of convergent and discriminant validity of the TEHS was also needed. In terms of convergent validity, we expected that the 6-item TEHS would be significantly positively related to a variety of measures, based on our prior studies and the existing literature (e.g., Morote et al., 2017; Vetter et al., 2018; Witvliet et al., 2019). First, we expected that it would relate positively to constructs previously shown to correlate with cognitive hope: resilience (Smith et al., 2008), flourishing (Diener et al., 2009), generosity (Smith and Hill, 2009), and happiness (Lyubomirsky and Lepper, 1999). Further, we expected that the TEHS would relate positively to sense of power (Anderson et al., 2012), given that sense of empowerment is theoretically related to hope (Snyder, 2002). In terms of discriminant validity, we expected that the TEHS would be significantly inversely related to stress (Cohen et al., 1983) and loneliness (Russell et al., 1980), which have been shown to negative correlate with cognitive hope in past studies (e.g., Feldman, 2021; Li et al., 2021). Finally, in this study we measured values (Lindeman and Verkasalo, 2005) and the “Big-Five” dimensions of personality (Gosling et al., 2003), but we did not make any *a priori* hypotheses about their relationships with the TEHS.

### Participants and procedures

Study participants were adults (*N* = 2,176) from around the world who voluntarily enrolled in a free massive open online course on happiness. Participants were primarily women (70.7%), White (52.8%), and nearly equally distributed in terms of marital status of being unmarried (37.6%) or married (35.1%). Participants in our sample were well educated (69.1% with bachelor’s degree or higher), mostly did not have any children (60.9%), and had a mean age of 37.06 (*SD* = 14.87). Participants were located in 193 different countries around the globe. While the United States was the most commonly reported country of residence (*N* = 650, 29.9%), the majority of the sample (70.1%) was from outside the United States. The top 15 countries of residence for our participants outside of the U.S. were: India (*N* = 140), Canada (*N* = 101), United Kingdom (*N* = 86), Australia (*N* = 75), Germany (*N* = 72), Brazil (*N* = 60), France (*N* = 49), Singapore (*N* = 39), Indonesia (*N* = 33), Mexico (*N* = 33), China (*N* = 28), Italy (*N* = 28), Netherlands (*N* = 26), Philippines (*N* = 26), and New Zealand (*N* = 24).

All course enrollees were invited to complete an optional pre-course survey, where they had the opportunity to provide informed consent and demographic information, and respond to a battery of questionnaires, described below.

### Measures

#### Trait emotion hope scale (TEHS)

Based on the findings from Study 2, here we administered the 6-item TEHS scale, which includes the following items: (1) In general, I feel filled with hope; (2) I feel hopeful about getting the things in life that are most important to me; (3) When things don’t go my way, I still feel hopeful, (4) Most days, I feel full of hope; (5) I feel hopeful when I think about my goals; (6) Even when I experience setbacks, I still feel hopeful. All items were rated on an 8-point Likert Scale (1 = *Definitely False*, 8 = *Definitely True*). The total TEHS score is derived by summing the six items (R: 6 – 48), where higher scores indicate greater hope. Internal consistency for the TEHS will be reported in the Results section.

#### Brief resilience scale (BRS)

Participants completed the same BRS (Smith et al., 2008) as Study 1. Internal consistency was α = 0.85.

#### Flourishing scale (FS)

The FS (Diener et al., 2009) is an 8-item measure of success in life areas such as relationships, self-esteem, purpose, and optimism. The scale provides a single psychological well-being score. Items are rated on a 7-point Likert scale (1 = *Strongly disagree*, 7 = *Strongly agree*) and are summed, with higher scores indicating greater psychological resources and strengths. Internal consistency was α = 0.90.

#### Interpersonal generosity scale (IGS)

The IGS (Smith and Hill, 2009) is a 10-item measure of the degree to which a person is generous with their attention, emotion, time, and energy with others. Items are rated on a 6-point Likert scale (1 = *Strongly Disagree*, 6 = *Strongly Agree*). Items are summed, with higher scores indicating greater generosity. Internal consistency was α = 0.90.

#### Perceived stress scale (PSS-4)

The PSS-4 (Cohen et al., 1983) is a 4-item brief version of the original PSS, which measures an individual’s perceptions of stress during the past month. Items are rated on a 5-point Likert scale (0 = *Never*, 4 = *Very Often*). All items are summed, with higher scores indicating more stress. Internal consistency was α = 0.75.

#### Sense of power scale (SPS)

The 8-item SPS (Anderson et al., 2012) assesses personal sense of power in general relationships with others. Items are rated on a 7-point Likert scale (1 = *Disagree strongly*, 7 = *Agree strongly*) and are summed, with higher scores indicating a greater sense of power. Internal consistency was α = 0.85.

#### Short schwartz value survey (SSVS)

The 10-item SVSS (Lindeman and Verkasalo, 2005) provides broad insight into 10 basic values (a single item per value): power, achievement, hedonism, stimulation, self-direction, universalism, benevolence, tradition, conformity, and security. Each value is rated on a 9-point Likert scale (0 = *Opposed to my values*, 8 = *Of supreme importance*). Because each item assesses a different value, internal consistency is not calculated.

#### Subjective happiness scale (SHS)

Participants completed the same SHS (Lyubomirsky and Lepper, 1999) as Study 1. Internal consistency was α = 0.88.

#### Ten-item personality inventory (TIPI)

The TIPI (Gosling et al., 2003) is a 10-item measure of the “Big-Five” personality dimensions of extraversion, agreeableness, conscientiousness, emotional stability, and openness. Rated on a 7-point Likert scale (1 = *Disagree strongly*, 7 = *Agree strongly*), each dimension is a subscale average of two items, with higher scores indicating higher levels of the personality dimension. According to the authors, the TIPI is appropriate to use when time is limited and personality is not the primary topic of interest. Per Gosling et al., 2003, given that there are only two items per scale, it is not meaningful to calculate internal consistency.

#### UCLA loneliness scale (ULS-4)

The 4-item ULS-4 (Russell et al., 1980) assesses loneliness. All items are rated on a 4-point Likert scale (1 = *Never*, 4 = *Often*). Higher scores indicate greater loneliness. Internal consistency was α = 0.75.

#### Demographics

Participants responded to a number of demographic questions, including age, gender, ethnicity, country of residence, education, marital, and parental status.

### Statistical analysis

We utilized Pearson’s correlations to assess convergent and discriminant validity. Basic descriptive statistics were characterized by means and standard deviations for all measures. Correlations and descriptive statistics were conducted in SPSS version 28 (IBM, 2021). Given that Study 3 was overpowered for correlations and that external validity is assessed via the magnitude of Pearson’s *r* and not statistical significance, *p*-values were not interpreted. While there is no universally agreed-upon interpretation of correlation coefficients, we used ranges that are often recommended for psychological research. We interpreted correlations of ± 0.30 and below as “weak,” correlations of ± 0.70 and above as “strong,” and correlations between these two values as “moderate” (Dancey and Reidy, 2007).

Given the ordinal nature of our data, a confirmatory factor analysis (CFA) using diagonally weighted least squares (DWLS) estimation was conducted (Li, 2016). The CFA was conducted in JASP Version 0.16.1 (JASP, 2022) and tested the single-factor model from Study 2. Root Mean Square Error of Approximation (RMSEA), Comparative Fit Index (CFI), and Standardized Root Mean Square Residual (SRMR) were used as fit indices. According to Kenny (2015), good fitting models are represented by an RMSEA less than 0.05, CFI above 0.95, and SRMR below 0.08.^3^

### Results

The 6-items of the TEHS had excellent internal consistency reliability, α = 0.94 (ω = 0.94). The range of corrected item-total correlations were from 0.77 to 0.85.

#### Confirmatory factor analysis

**FIGURE 2:**
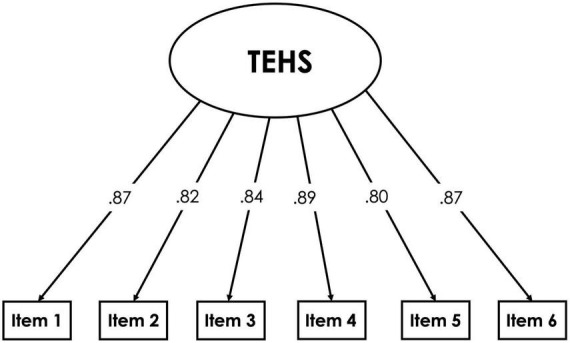
Graphical representation of confirmatory factor analysis (CFA) model.

As mentioned, the CFA assessed the fit of a single-factor model. Figure 2 contains a graphical representation of the model. This model demonstrated excellent fit (RMSEA = 0.039, CFI = 0.997, and SRMR = 0.037). All items loaded strongly on the single factor, with loadings ranging from 0.80 to 0.89.

#### Convergent and discriminant validity

Table 7 contains means, standard deviations, and correlations. The TEHS had positive, strong correlations with happiness (*r* = 0.73) and flourishing (*r* = 0.71); positive, moderate correlations with resilience (*r* = 0.57), sense of power (*r* = 0.41), emotional stability (*r* = 0.49), conscientiousness (*r* = 0.31), and openness (*r* = 0.31); and positive, weak correlations with generosity (*r* = 0.25), extraversion (*r* = 0.29), and agreeableness (*r* = 0.24). Single-item measures of the following values were also positively yet very weakly correlated with TEHS scores: self-direction (*r* = 0.18), tradition (*r* = 0.17), conformity (*r* = 0.16), benevolence (*r* = 0.15) stimulation (*r* = 0.13), universalism (*r* = 0.13), achievement (*r* = 0.11), and security (*r* = 0.10). The TEHS had negative, moderate correlations with stress (*r* = −0.55) and loneliness (*r* = 0.49). Finally, the TEHS had no relationship with single-item value measures of power (*r* = 0.03) or hedonism (*r* = −0.01).

**TABLE 7:** Study 3 means, standard deviations, and bivariate correlations.

Variables	TEHS 6-item r, p	M (SD)
TEHS – 6-item	− −	33.83 (9.21)
BRS	0.57**	3.12 (0.79)
FS	0.71**	41.51 (8.34)
IGS	0.25**	46.50 (8.41)
PSS-4	-0.55**	8.37 (3.15)
SOPS	0.41**	4.45 (0.66)
SSVS – *Power*	0.03	3.94 (2.12)
SSVS – *Achievement*	0.11**	5.53 (1.91)
SSVS – *Hedonism*	-0.01	4.92 (2.17)
SSVS – *Stimulation*	0.13**	5.14 (1.95)
SSVS – *Self-direction*	0.18**	6.50 (1.53)
SSVS – *Universalism*	0.13**	6.45 (1.66)
SSVS – *Benevolence*	0.15**	6.77 (1.41)
SSVS – *Tradition*	0.17**	4.62 (2.22)
SSVS – *Conformity*	0.16**	4.53 (2.22)
SSVS – *Security*	0.10**	5.66 (1.86)
SHS	0.73**	4.48 (1.40)
TIPI – *Extraversion*	0.29**	3.94 (1.52)
TIPI – *Agreeableness*	0.24**	4.92 (1.12)
TIPI – *Conscientiousness*	0.31**	5.10 (1.28)
TIPI – *Emotional Stability*	0.49**	4.08 (1.42)
TIPI – *Openness*	0.31**	5.35 (1.11)
ULS-4	-0.49**	9.34 (2.58)

** = *p* < 0.01.

### Brief discussion

The CFA results from Study 3 confirm the fit of the single-factor model in a large, multinational sample. Convergent validity analyses suggest that emotional hope is strongly related to happiness and flourishing. Additionally, three of the big-five personality traits (emotional stability, conscientiousness, and openness) were moderately related to the TEHS, with extraversion approaching the cutoff of 0.30 with a correlation of 0.29. The TEHS was moderately inversely related to negative affective states such as stress and loneliness. The TEHS does not appear to have a relationship with values. Together, these findings highlight that the TEHS is uniquely associated with emotional hope.

## General discussion

In this series of studies, we sought to develop a measure of the theorized “emotion set” in Snyder’s (2002) Elaborated Hope Theory. Specifically, across three studies (*N* = 2,900), we set out to assess the psychometric properties the Trait Emotion Hope Scale (TEHS). The TEHS was initially developed using 15 emotional hope items; however, an EFA (Study 2) and CFA (Study 3) revealed a stable single-factor structure comprised of 6-items. When examining internal consistency reliability and construct validity, the TEHS performed very well across all three studies. When examining the unique variance accounted for by the TEHS (Study 2), the TEHS accounted for significant unique variance beyond cognitive hope (i.e., the Adult Hope Scale; Snyder et al., 1991), indicating that the two constructs are indeed distinct. This research has important implications for Hope Theory as well as for future empirical research.

As mentioned previously, Elaborated Hope Theory (Snyder, 2002) asserts that the trait-like components of hope consist of both a cognitive and emotion set. Unfortunately, until now, measures related to Hope Theory have largely focused on the model’s cognitive aspects. The TEHS is the first instrument to explicitly assess Snyder’s (2002) “emotion set,” which opens the door for future researchers to test the complete elaborated hope model. In short, the TEHS offers a tool by which Hope Theory researchers may gain a more complete understanding of hope as a multifaceted phenomenon, incorporating both cognition and emotion.

While the present research makes important theoretical and empirical contributions, it is not without limitations, which opens up exciting avenues for future research. First, the present research did not examine whether emotional hope, as measured by the TEHS, predicts important behavioral outcomes such as actual goal attainment. While past research has found this to be the case for cognitive hope (Feldman et al., 2009), it is unknown how emotional hope relates to goal attainment. Thus, future research using the TEHS should examine whether it, as compared to cognitive measures of hope, uniquely predicts an individual’s likelihood of attaining goals. For example, given the relationship between emotions and behavior (Frijda, 1986; Izard and Ackerman, 2000; Schwarz and Clore, 2007), and the link between emotion and goal-pursuit (Oatley and Johnson-Laird, 1987), researchers could test whether greater emotional hope helps an individual behave in such ways that make attainment of goals more likely, above and beyond the effects of having hopeful cognitions. Relatedly, greater trait emotional hope may increase the likelihood of attaining certain goals, such as those that are more affective in nature, compared to goals that are less affective in nature.

Additionally, while we made an effort to examine the TEHS in diverse samples including young adults (Study 1), middle-aged adults (Study 2 and 3), and adults from around the globe (Study 3), this research is primarily comprised of well-educated women. Given that the experience of emotions, such as hope, may differ based on a person’s identity and life experiences, future research would greatly benefit from examining the TEHS (and future translations of the scale) in more diverse populations that are more representative, including a wide variety of genders, ethnicities, levels of education, and socioeconomic statuses. Future research can also test for factorial invariance across cultures. Relatedly, the present research only examined the TEHS in adults. Given the face-validity of the six TEHS items, we believe it could be reasonably used with adolescents, however, the appropriateness of the TEHS with younger populations should be explicitly tested.

Finally, it is important to note that the three studies reported in this paper utilized a cross-sectional design. Thus, the regression analyses conducted cannot be interpreted through a causal relationship lens. Because predictive validity of the TEHS cannot be established through a cross-sectional design, it will be important for future research to utilize longitudinal study designs where temporal precedence can be established.

In summary, this sequence of studies has demonstrated that our 6-item hope scale, the TEHS, has sound psychometric properties and provides a valid measure for empirical testing hope as an emotion. We believe that the use of this short 6-item scale will permit a greater understanding of individual variations in hope that exist beyond the traditional cognitive factors. We foresee that the TEHS will assist researchers in gaining greater specificity and further understanding this important and complex construct.

## Data availability statement

The raw data supporting the conclusions of this article will be made available by the authors, without undue reservation.

## Ethics statement

The studies involving humans were approved by the Santa Clara University and the University of California, Berkeley (Study 3) Institutional Review Boards. The studies were conducted in accordance with the local legislation and institutional requirements. The participants provided their written informed consent to participate in this study.

## Author contributions

DF: Conceptualization, Data curation, Formal analysis, Investigation, Methodology, Project administration, Software, Visualization, Writing – original draft, Writing – review & editing. HJ: Conceptualization, Data curation, Formal analysis, Funding acquisition, Investigation, Methodology, Project administration, Software, Visualization, Writing – original draft, Writing – review & editing.

## Footnotes

1 Following the suggestion of our reviewers, we also conducted an EFA with parallel analysis in JASP (2022) which also supported a single factor (the second potential factor that emerged included all six of the reverse coded items, which accounted for an additional 4.1% of the variance).

2 The Cronbach’s Alpha if item was deleted statistic suggested that the scale could not be further improved beyond this. The range of corrected item-total correlations were from 0.77 to 0.90.

3 We intentionally did not include the chi-square statistic. Chi-square is often not utilized in CFA when sample sizes are very large because it can lead to rejecting models that are, in fact, good fits to the data (see Schermelleh-Engel et al., 2003).
